# Comparison of complete multi-level vs. iliac-only revascularization for concomitant iliac and superficial femoral artery occlusive disease

**DOI:** 10.3389/fsurg.2023.1188990

**Published:** 2023-05-25

**Authors:** Hong Cheng Ren, Tian Run Li, Jin Man Zhuang, Xuan Li, Jing Yuan Luan, Chang Ming Wang, Ming Chao Ding

**Affiliations:** ^1^ Department of Intervention Vascular, Aerospace Center Hospital, Beijing, China; ^2^ Department of Intervention Vascular Surgery, Peking University Third Hospital, Beijing, China

**Keywords:** deep femoral artery, superficial femoral artery, multi-level peripheral artery disease, peripheral arterial disease, home-based walking exercise

## Abstract

**Objective:**

The aim of this study is to compare the efficacy and safety of complete multi-level vs. iliac-only revascularization for the treatment of concomitant iliac and superficial femoral artery (SFA) occlusive disease.

**Methods:**

A total of 139 consecutive adult patients with severe stenosis and occlusive iliac and SFA disease with Rutherford categories 2–5 underwent multi-level (*n* = 71) and iliac-only (*n* = 68) revascularization at the Department of Intervention Vascular Surgery, Peking University Third Hospital, and Aerospace Center Hospital, between March 2015 and June 2017. Improvement in Rutherford class, perioperative major adverse events, the length of stay, survival rate, and limb salvage rate were assessed. The neutrophil–lymphocyte ratio and platelet–lymphocyte ratio were compared between the two groups.

**Results:**

At 48 months, improvement in the Rutherford category was observed in the two groups with no significant difference (*P* = 0.809). Additionally, the two groups were similar concerning the primary patency (84.0% vs. 79.1%, *P* = 0.717) and limb salvage rate (93.1% vs. 91.3%, *P* = 0.781). A higher proportion of the perioperative major adverse events (33.8% vs. 27.9%, *P* = 0.455), the all-cause mortality (11.3% vs. 8.8%, *P* = 0.632), and the average length of hospital stay [7.0 (6.0, 11.0) vs. 7.0 (5.0, 8.0), *P* = 0.037] were seen in the multi-level group compared with the iliac-only group.

**Conclusion:**

For concomitant iliac and superficial femoral artery occlusive disease, iliac-only revascularization has favorable efficacy and safety outcomes compared with complete multi-level revascularization in selected patients with patent profunda femoris artery and at least one healthy outflow tract of the infrapopliteal artery.

## Highlights

• Type of research: retrospective two-center cohort study.
• Key findings: In 139 patients with concomitant iliac and superficial femoral artery occlusive disease, no difference between multi-level vs. iliac-only revascularization was detected with respect to Rutherford category, primary patency, limb salvage, survival rate, and the perioperative major adverse events. Iliac-only revascularization showed better performance in the length of stay.
• Take-home message: This study suggests that iliac-only revascularization has favorable efficacy and safety outcomes compared with multi-level revascularization in selected patients.
• Table of contents summary: In this retrospective cohort study of 139 patients, for concomitant iliac and superficial femoral artery occlusive disease, iliac-only revascularization has favorable efficacy and safety outcomes compared with complete multi-level in selected patients with patent profunda femoris artery (PFA) and at least one healthy outflow tract of the infrapopliteal artery.

## Introduction

Patients with diffuse multi-level peripheral arterial disease have achieved disappointing results following complete surgical or endovascular revascularization (1–3) and account for approximately 30% (4). Iliac-only revascularization strategy (5) can improve collateral circulation, via the patent profunda femoris artery (either pre-existing or re-established by endovascular intervention), to the ischemic lower limb in cases of both iliac artery and superficial femoral artery (SFA) occlusive disease. On the one hand, the deep femoral artery (DFA), which acts as a collateral pathway, plays a critical role in the irrigation of the limbs when the SFA is severely stenosized or occluded (6). On the other hand, the iliac artery and DFA lesion are less susceptible to atherosclerosis than the SFA lesion in patients with diffuse multilevel peripheral arterial disease (PAD), and prior studies have demonstrated that endovascular treatments have a greater long-term primary patency for the iliac artery and DFA lesion compared with SFA lesion (7–9). Therefore, SFA revascularization is not always justified in multifocal arterial disease, as some patients can solely achieve symptom relief and limb salvage by using the iliac-only revascularization strategy (5, 6, 10). However, for the treatment of concomitant iliac and SFA occlusive disease, there has been a few published discussions about the complete multi-level vs. iliac-only revascularization. Considering the above knowledge, the objective of this study is to compare the efficacy and safety of complete multi-level vs. iliac-only revascularization for the treatment of concomitant iliac and SFA occlusive disease.

## Methods

This retrospective study was approved by the hospital's medical ethics committee, and a written informed consent was obtained from all eligible patients. A total of 139 consecutive adult patients with concomitant iliac and SFA occlusive disease with Rutherford classes of 2–5 underwent multi-level and iliac-only revascularization at the Department of Intervention Vascular Surgery, Peking University Third Hospital, and Aerospace Center Hospital, between March 2015 and June 2017. Patients’ data, such as demographics, comorbidities, lesion characteristics, procedural details, and outcome variables, were collected from the hospital’s electronic medical records. The patients were required to have the patent profunda femoris artery (either pre-existing or re-established by endovascular intervention) and at least one healthy outflow tract of the infrapopliteal artery. The exclusion criteria were patients with acute limb ischemia or anastomosis stenosis, limited life expectancy or limited walking, pregnancy, or lactation. The intervention strategies were based on the surgeon's preference and patients’ selection. The details of the endovascular procedures were obtained from the electronic patient records. The lesion characteristics were evaluated by the Trans-Atlantic Inter-Society Consensus II classification.

### Multi-level revascularization

Standard endovascular maneuvers with diagnostic angiography were performed to assess the location and specific types of lesions. Typical or hydrophilic guidewires and catheters were then used to cross both the iliac artery and the SFA lesion. Pre-dilatation was performed with the semi- or non-compliant conventional balloon-size step-up method, followed by the inflation time of 180 s. The length of the balloon had to be sufficient to cover at least 1 cm of the lesion both distally and proximally. Self-expanding stents were implanted in cases with flow-limiting dissection and residual stenosis of >30%. Post-dilation was performed using balloons with a nominal diameter equal to that of the implanted devices.

### Iliac-only revascularization

Standard endovascular maneuvers with percutaneous transluminal angioplasty and self-expanding stents were systematically performed for iliac artery lesions to secure direct blood flow to the DFA. The patent profunda femoris artery was pre-existing or re-established through endovascular intervention, and no concomitant treatment for SFA lesions was performed. In addition, all patients were instructed to conduct home-based walking exercise at least three sessions for at least 30 min every week.

### Pharmacological therapy

Pre-procedural antiplatelet therapy included clopidogrel (75 mg/day) and aspirin (100 mg/day) 3 days before the procedure. Low-molecular-weight heparin was administered 3 days after surgery to maintain an activated coagulation time of approximately 70–90 s. Dual antiplatelet therapy after the procedure was prescribed for 1 year, followed by single antiplatelet therapy with clopidogrel (75 mg/day) per life.

### Study follow-up and assessment definitions

Patients were followed up before discharge; at 1, 3, and 6 months through regular visits; and annually thereafter. The outcome measures of effectiveness were assessed by primary patency at 48 months, improvement in Rutherford category, and limb salvage rate. Primary patency was defined as without any reintervention of the target lesion to maintain patency. The safety endpoints were perioperative major adverse events, all-cause mortality, and the average length of hospital stay. Differences in neutrophil–lymphocyte ratio and platelet–lymphocyte ratio were also calculated.

### Statistical analysis

All statistical analyses were performed with SPSS (version 24.0 for Windows, SPSS Inc., Chicago, IL, United States) and plotted with GraphPad Prism 8.0 (GraphPad, San Diego, CA, United States). A probability rate of <0.05 was statistically considered significant. The quantitative data were expressed as means and SDs for normally distributed variables and medians (quartiles) for non-normally distributed variables and were assessed by *t*-test and Mann–Whitney *U* test, respectively. Qualitative data were described as proportions and compared using the chi-square or Fisher's exact tests. Primary patency, limb salvage, and survival rates were determined with the Kaplan–Meier method and differences with the log-rank test.

## Results

### Baseline characteristics of the patient and the lesion

A total of 139 adult patients (71 in the multi-level group and 68 in the iliac-only group) were observed based on the inclusion/exclusion criteria between March 2015 and June 2017. The multi-level group included 16 females (with mean age of 68.79 ± 9.30 years), while the iliac-only group included 11 females (with mean age of 68.49 ± 8.79 years). The two groups were comparable at baseline patient and lesion characteristics (Tables 1 and 2).

**Table 1:** Baseline patient characteristics^a^.

Variables	Multi-level	Iliac-only	Statistics	*P*-value
	(*n* = 71)	(*n* = 68)	(Z/t/*χ*2)	
Age, years	68.79 ± 9.30	68.49 ± 8.79	−0.268	0.789
Sex			0.897	0.344
Female	16 (22.5%)	11 (16.2%)		
Male	55 (77.5%)	57 (83.8%)		
BMI (kg/m^2^)	24.38 ± 3.73	23.67 ± 3.06	1.226	0.222
Smoking history	31 (46.5%)	41 (60.3%)	2.663	0.103
Diabetes mellitus	36 (50.7%)	35 (51.5%)	0.008	0.928
Hypertension	50 (70.4%)	54 (79.4%)	1.490	0.222
Dyslipidemia	26 (36.6%)	28 (41.2%)	0.304	0.582
Coronary artery disease	17 (23.9%)	21 (30.9%)	0.842	0.359
Cerebrovascular accident	18 (25.4%)	17 (25.0%)	0.002	0.962
NLR	3.3 (2.3,9.2)	2.7 (1.0,3.9)	−0.105	0.196
PLR	142.4 ± 68.4	108.7 (90.5,176.2)	−1.152	0.249
Baseline Rutherford category			1.613	0.656
2	7 (9.9%)	10 (14.7%)		
3	30 (42.2%)	30 (44.1%)		
4	26 (36.6%)	19 (27.9%)		
5	8 (11.3%)	9 (13.3%)		

BMI, body mass index; NLR, neutrophil−lymphocyte ratio; PLR, platelet−lymphocyte ratio.

^a^ Normally distributed data were described as mean ± SD, whereas skewed data as medians (quartiles). The qualitative data were described as counts (percentage).

**Table 2:** Baseline lesion characteristics^a^.

Variables	Multi-level	Iliac-only	Statistics	*P*-value
	(*n* = 71)	*n* = (68)	t/χ2	
Iliac lesion TASC			4.772	0.189
A	34 (47.9%)	45 (66.2%)		
B	30 (42.3%)	19 (27.9%)		
C	5 (7.0%)	3 (4.4%)		
D	2 (2.8%)	1 (1.5%)		
Common femoral artery	3 (4.2%)	3 (4.4%)	0.003	0.957
SFA lesion TASC			5.933	0.115
A	10 (14.1%)	8 (11.8%)		
B	16 (22.5%)	7 (10.2%)		
C	27 (38.0%)	25 (36.8%)		
D	18 (25.4%)	28 (41.2%)		
Deep femoral artery	8 (11.3%)	12 (17.6%)	1.148	0.284
Popliteal artery disease	23 (32.4%)	19 (27.9%)	0.327	0.568
Runoff vessels			1.121	0.571
1	28 (39.4%)	21 (30.9%)		
2	28 (39.4%)	31 (45.6%)		
3	15 (21.2%)	16 (23.5)		

SFA, superficial femoral artery; TASC, trans-atlantic inter-society consensus.

^a^ The quantitative data were expressed as means and SDs. The qualitative data were described as counts (percentage).

### Effectiveness outcomes

**Figure 1:**
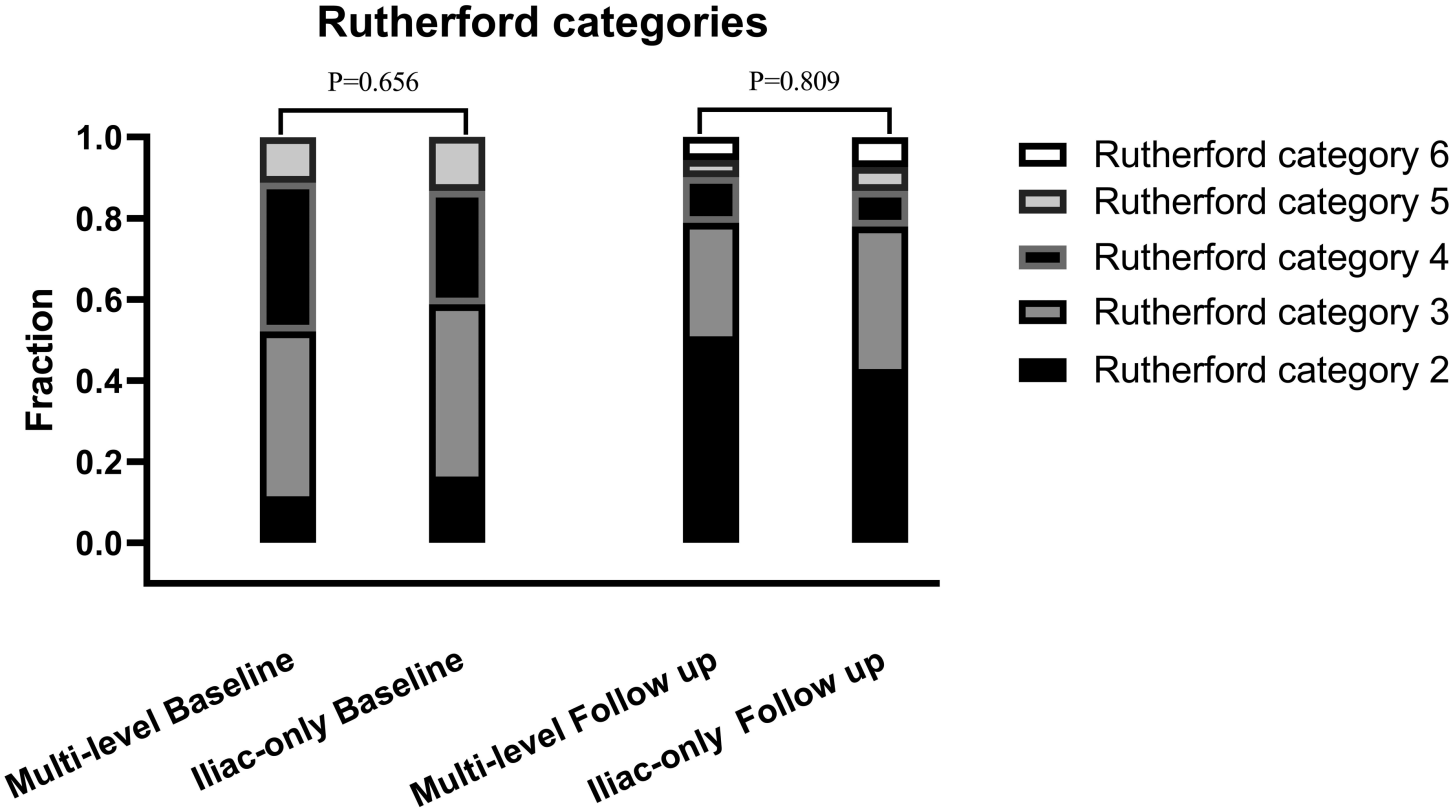
Rutherford clinical category at baseline and follow-up.

**Figure 2:**
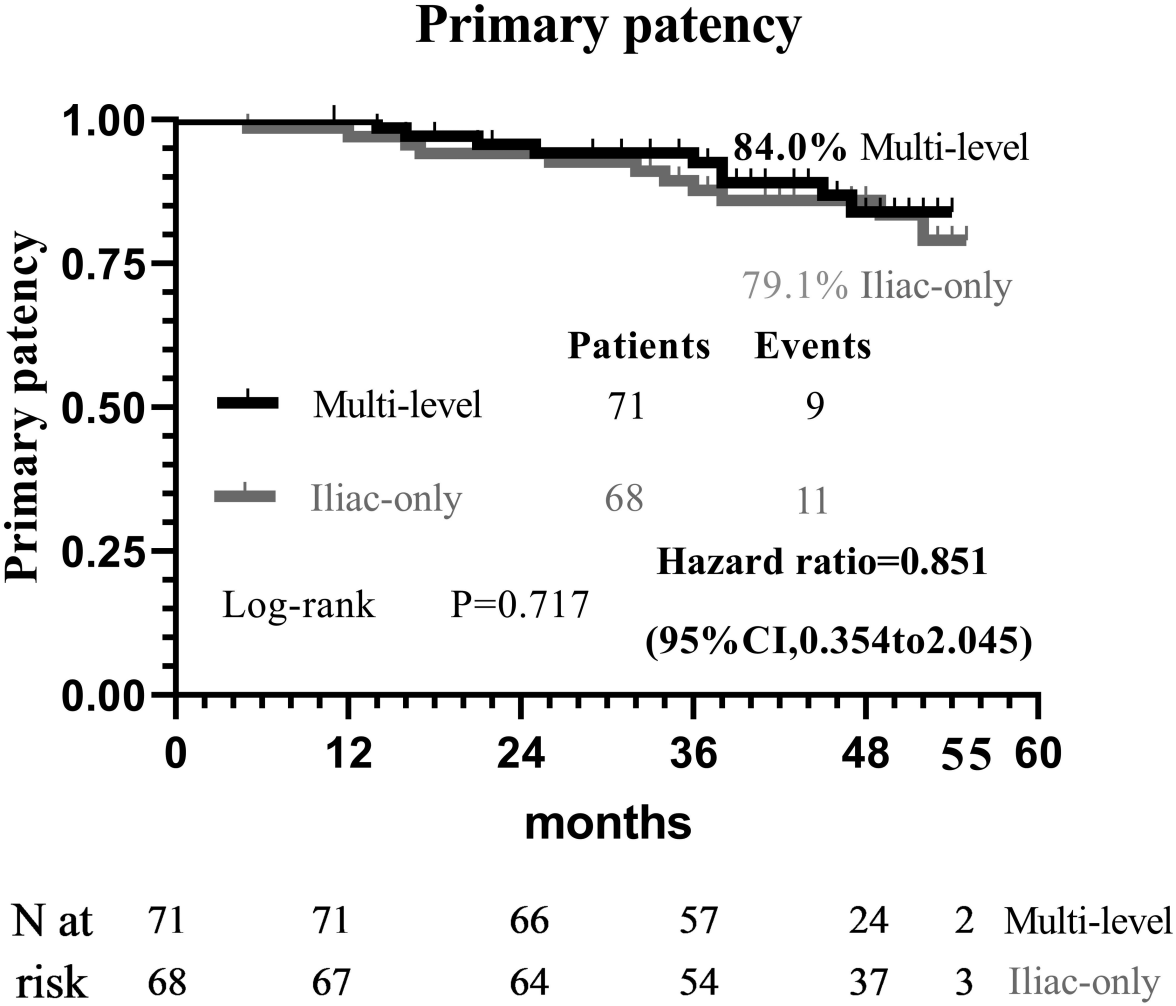
Kaplan–Meier curves of primary patency.

**Figure 3:**
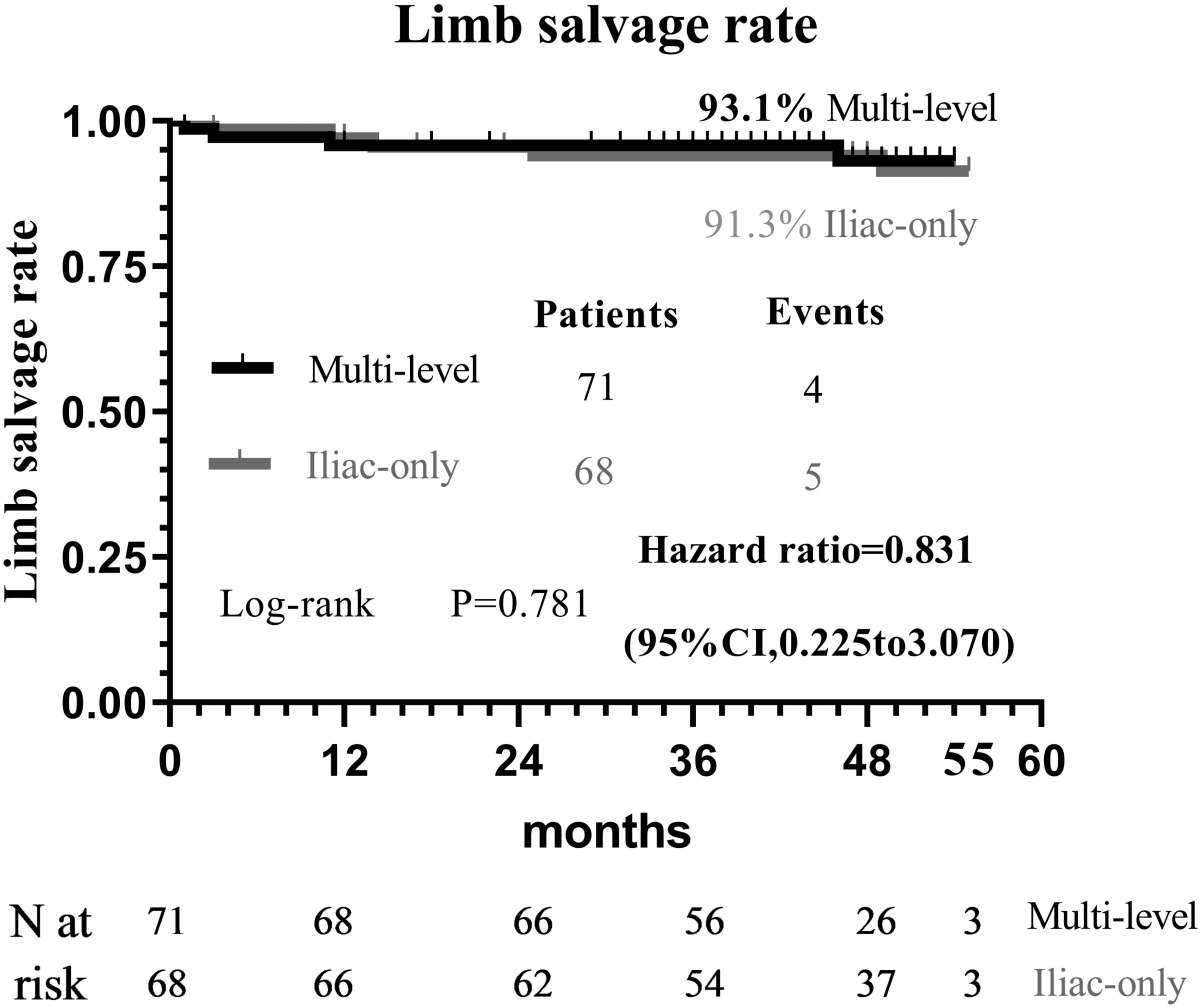
Kaplan–Meier curves of limb salvage rate.

**Figure 4:**
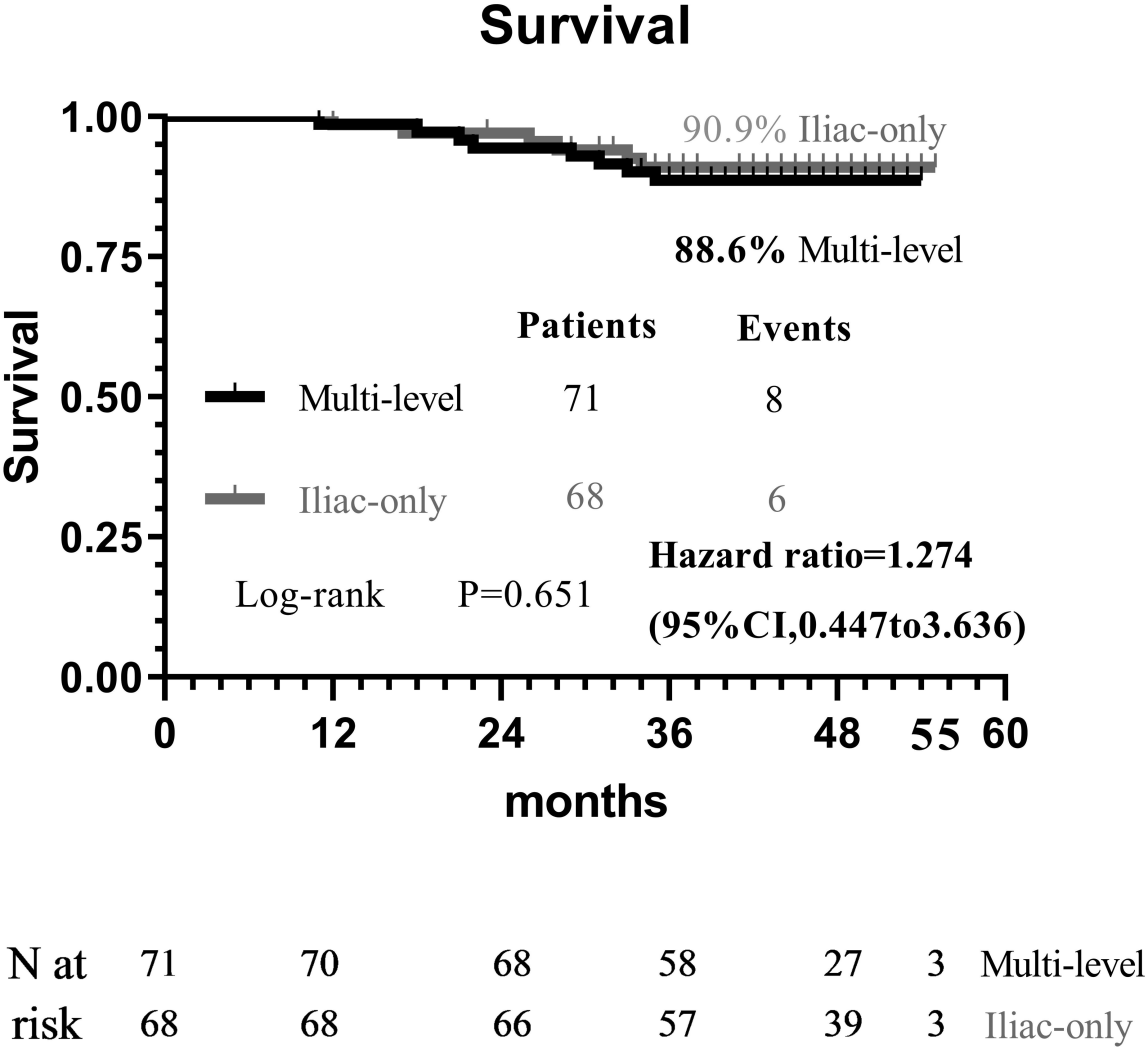
Kaplan–Meier curves of survival.

At 48 months, an improvement in Rutherford category was observed in the two groups, and no significant difference was detected (*P* = 0.809, Figure 1). Moreover, the two groups were similar concerning primary patency (84.0% vs. 79.1%, *P* = 0.717, Figure 2), limb salvage rate (93.1% vs. 91.3%, *P* = 0.781, Figure 3), and survival rates (90.9% vs. 88.6%, *P* = 0.651, Figure 4).

### Safety outcomes

A higher proportion of the perioperative major adverse events (33.8% vs. 27.9%, *P* = 0.455), the all-cause mortality (11.3% vs. 8.8%, *P* = 0.632), and the length of hospital stay [7.0 (6.0, 11.0) vs. 7.0 (5.0, 8.0), *P* = 0.037] were seen in the multi-level group compared with the iliac-only group (Table 3).

**Table 3:** Safety outcomes^a^.

	Multi-level	Iliac-only	t/χ^2^	*P*-value
	(*n* = 71)	*n* = (68)		
Major adverse events	24 (33.8%)	19 (27.9%)	0.559	0.455
Myocardial infarction	0 (0.0%)	1 (1.5%)	—	1.000
Cardiac insufficiency	2 (2.8%)	2 (3.0%)	0.002	0.965
Cerebrovascular stroke	1 (1.4%)	1 (1.5%)	0.001	0.975
Pulmonary complications	1 (1.4%)	2 (3.0%)	0.386	0.534
Renal complications	0 (0.0%)	1 (1.5%)	—	1.000
Hematoma	3 (4.2%)	1 (1.5%)	0.943	0.331
All-cause mortality	8 (11.3%)	6 (8.8%)	0.229	0.632
Amputation	4 (5.6%)	5 (7.4%)	0.170	0.681
Thrombosis	3 (4.2%)	0 (0.0%)	—	1.000
Infection	1 (1.4%)	0 (0.0%)	—	1.000
Dissection	1 (1.4%)	0 (0.0%)	—	1.000
Length of hospital stay	7.0 (6.0,11.0)	7.0 (5.0,8.0)	−2.090	0.037

^a^ Normally distributed data were described as mean ± SD, whereas skewed data as medians (quartiles). The qualitative data were described as counts (percentage).

## Discussion

**Figure 5:**
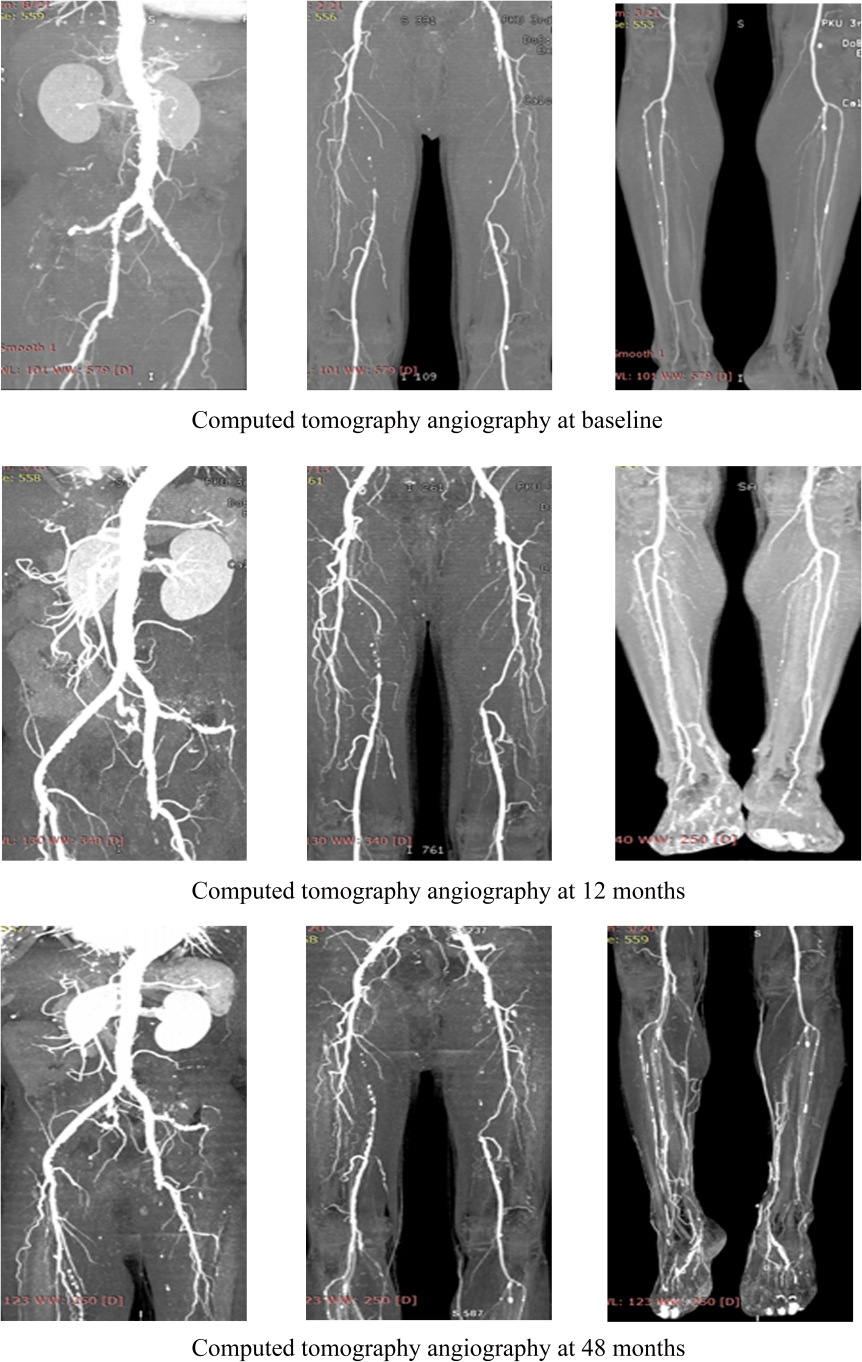
Computed tomography angiography at baseline and follow-up. A 63-year-old male with severe stenosis and occlusive iliac and SFA disease with Rutherford category 3 underwent iliac-only revascularization in December 2015. During follow-up, improvement in the Rutherford categories (3 to 2) and the collateral vessels were observed. The patency of the iliac stent was freedom from significant restenosis or occlusion without any reintervention.

For patients with concomitant iliac and SFA occlusive disease, vascular surgeons are probably reluctant to perform a complete multi-level reconstruction in light of the prolonged operation time, operative complication, or poor prognosis (11). In another way, revascularization from an iliac artery to the DFA without repair of the SFA has been performed to achieve relatively good results through either surgical or endovascular treatment (5, 6, 10–13). To the best of our knowledge, no comparative data were found regarding the relative efficacy of complete multi-level vs. iliac-only revascularization. In the present study, the improvement in Rutherford category (*P* = 0.809) and the limb salvage rate (93.1% vs. 91.3%, *P* = 0.781) were similar between the two groups. This indicated that the improvement of conditions, for the patients with both iliac and SFA lesions, and the presence of at least one healthy outflow tract of the infrapopliteal artery were observed in the majority of patients through either complete multi-level or iliac-only revascularization. A possible explanation is that the blood flow through the isolated patent DFA in the iliac-only group was equal to the flow through both patent SFA and DFA in the multi-level group (14). Furthermore, home-based walking exercise might accelerate the blood restoration of chronic critical limb ischemia and promote the formation of collateral vessels in SFA occlusive disease (15, 16). Therefore, multi-level revascularization is not always justified in patients with iliac and SFA lesions, considering that in most of these patients iliac-only intervention is required (Figures 5).

In theory, poor runoff is one of the main risk predictors of iliac arterial stent failure. Mousa et al. (17) advocate a hybrid therapy of combined percutaneous iliac angioplasty and infrainguinal surgical reconstruction in patients with multisegmental arterial occlusive disease. However, Timaran et al. (2) failed to demonstrate that concomitant infrainguinal arterial revascularization improves the patency of the iliac stent. Kudo et al. (18) reported that angioplasty for occluded SFA disease did not improve primary patency and clinical outcomes following percutaneous transluminal angioplasty of the iliac arteries. The patency of the iliac artery stent (84.0% vs. 79.1%, *P* = 0.717) was comparable between the two groups. The results suggest that SFA patency does not adversely interfere with the primary failure of endovascular intervention for iliac arterial disease. Similarly, de Athayde Soares et al. (6) evaluated the importance of SFA and PFA after endovascular treatment of chronic aortoiliac occlusive disease, and the PFA in conjunction with its collateral circulation may have a great value in primary patency (80.2% vs. 82.3%; *P* = 0.80) and limb salvage rate (91.3% vs. 86.1%; *P* = 0.60) after endovascular aorta-iliac intervention compared with patent SFA. Therefore, it is not indispensable to simultaneously cope with iliac and SFA lesions to prevent iliac stent failure.

The SFA is the site of common atherosclerotic lesion involvement, and endovascular strategies are the recommended first-line choice (19). At present, nevertheless, there are still many challenges in the revascularization of complex SFA disease, especially considering the long lesion, chronic total occlusion, heavy calcium, and in-stent restenosis. Through the advent of a new conception with “leaving nothing behind” and the application of a drug-coated device, the efficacy and safety of endovascular interventions are less robust in complex SFA disease which concerned about the failure of the provisional stenting rate as high as 40%–46% (20, 21) and increased all-cause mortality (22). Higher levels of the neutrophil–lymphocyte ratio before surgery, reflecting the severity of peripheral arterial disease, were associated with poor outcomes after lower extremity procedures (23). No significant differences in the neutrophil–lymphocyte ratio and the platelet–lymphocyte ratio were observed in the two groups. Symptoms sometimes worsen after angioplasty and SFA stents. The complete multi-level revascularization group is associated with higher overall perioperative major adverse events (33.8% vs. 27.9%, *P* = 0.455) and length of hospital stay [7.0 (6.0, 11.0) vs. 7.0 (5.0, 8.0), *P* = 0.037]. A possible interpretation is that SFA is one of the most hostile vascular environments among the human body and vascular endothelium damage inevitably caused by endovascular treatments, associated with the occurrence of perioperative complications and vessel restenosis and unsatisfactory clinical benefit. Another explanation is that the coverage of collateral pathways possibly deteriorates clinical outcome after failed endografts.

There were several limitations in the study. First, the retrospective observational study with potential bias affected the results of this trial. Second, data on pharmacological and pain management were not completely noted in both groups, particularly in participants with limb-threatening ischemia; thereby it may affect the strength of the conclusions. Third, the present study did not compare the ankle–brachial index between the multi-level and iliac-only groups. Nonetheless, Pandey et al. (24) suggest that ankle–brachial index is probably not a decision-making tool for selecting therapeutic strategies but an appropriate screening tool to detect patients with lower-extremity peripheral artery disease.

## Conclusion

For concomitant iliac and SFA occlusive disease, iliac-only revascularization has favorable efficacy and safety results compared with complete multi-level revascularization in selected patients with patent profunda femoris artery and at least one healthy outflow tract of the infrapopliteal artery.

## Data Availability

The original contributions presented in the study are included in the article, further inquiries can be directed to the corresponding authors.
